# Defining Outcomes Following Distal Radius Fractures: Correlation of Function, Pain, and Hand Therapy Utilization

**DOI:** 10.7759/cureus.8718

**Published:** 2020-06-20

**Authors:** Omar Sh Ahmed, Gabriela Cinotto, Daniel Boczar, Maria T Huayllani, Stephen D Trigg, Antonio J Forte, Kimberly McVeigh

**Affiliations:** 1 Cancer Clinical Studies Unit, Mayo Clinic Florida, Jacksonville, USA; 2 Plastic Surgery, Mayo Clinic Florida - Robert D. and Patricia E. Kern Center for the Science of Health Care Delivery, Jacksonville, USA; 3 Plastic Surgery, Mayo Clinic Florida, Jacksonville, USA; 4 Orthopaedics, Mayo Clinic Florida - Robert D. and Patricia E. Kern Center for the Science of Health Care Delivery, Jacksonville, USA; 5 Physical Medicine and Rehabilitation, Mayo Clinic Florida, Jacksonville, USA

**Keywords:** distal radius, fracture, hand therapy, dash, rom, vas

## Abstract

Background

Distal radius fractures (DRF) is one of the most common fractures in clinical practice. Our objective was to study the role of early hand therapy and its impact on pain and return to daily activities.

Methods

The charts of patients with DRFs seen between January 2016 and November 2017 in the Hand Center of Mayo Clinic Florida were reviewed retrospectively. Forty-nine patients with DRFs who met inclusion criteria were included in the analysis. The variables collected included: age, gender, side of the fracture, surgery vs non-surgery, time to start hand therapy, number of visits, shortened disabilities of the arm, shoulder, and hand (QuickDASH) initial and discharge scores, and visual analog scale (VAS) initial and discharge.

Results

The patients’ mean age was 67.90 years, (standard deviation (SD) 14.54), 38 (77.6%) were female, 28 (57.1%) had a right DRF, 21 (42.9%) had a left DRF, 38 (77.6%) had no surgery, 11 (22.4%) had surgery. The mean time from fracture to therapy is 32.41, (SD 24.13) days, and the mean total number of visits is 6.20 (SD 3.49). We noticed a statistically significant difference between the initial QuickDASH (59.27, SD 16.93) compared to the discharge QuickDASH (24.08, SD 12.77) (P-value <.001); and initial VAS (3.57, SD 1.71) with a discharge VAS (1.33, SD 0.97) (P-value <.001).

Conclusion

This retrospective study found a statistically significant reduction in the QuickDASH and VAS scores after six hand therapy visits. The results suggest that early rehabilitation interventions lead to improvements in pain and return to daily activity following DRF.

## Introduction

Distal radius fracture (DRF) is the most commonly found fracture in medical emergencies, especially among patients who fall with an extended wrist. For younger patients, these fractures are most often the result of high energy traumas, such as falling from a height and an automobile accident. In the elderly population, due to the osteoporosis-induced low bone density, these fractures often result from low energy traumas [1]. The annual incidence between sexes was estimated to be approximately 105/100,000 in men and 416/100,000 in women, with a ratio of women to men of 4:1 [2]. Furthermore, DRF is a condition that affects the population in general and is found in different sexes, races, ages, professional occupations, and socioeconomic levels.

The treatment of each fracture is selected according to the degree of injury and the possible involvement of adjacent structures. Flat, extra-articular lesions without deviations and non-comminution will be treated in a closed manner, with closed reduction and immobilization. Intra-articular fractures, exposed fractures, comminuted fractures, and deviated fractures are complex fractures and require surgical intervention. Tenorrhaphy and neurorrhaphy are commonly performed in complex forms of DRF when it reaches structures adjacent to the radius [1]. 

The complications of early and late DRFs are reflected in higher scores for the shortened disabilities of the arm, shoulder, and hand (QuickDASH), limitations of range of motion (ROM), higher visual analog scale (VAS) scores, carpal tunnel syndrome (CTS), stiffness of the fingers, complex regional pain syndrome (CRPS), ligament and tendon injury, arthrosis, hematoma, neurotmesis, infection, compartment syndrome [3,4]. 

Regardless of the type of fracture and treatment, DRF patients are often referred for hand therapy to achieve rapid recovery, strength, ROM improvement, and long-term disability reduction [5,6]. These goals are achieved by the involvement of an experienced certified hand therapist. Since DRFs may lead to psychological stress and physical disabilities that jeopardize the independence and productivity of these patients, hand therapists have an important role in recovery outcomes [7,8].

The aim of this retrospective study is to understand the impact in surgical outcomes of the early initiation of hand therapy, in a tertiary medical center, measured by VAS and QuickDASH.

## Materials and methods

Data source 

This retrospective review was approved by the Institutional Review Board (IRB: 17-010437) of Mayo Clinic and informed consent was waived due to minimal risk. The only potential risk was privacy, which was addressed by using only the medical records of subjects. The medical charts of all patients with DRFs and seen in Mayo Clinic Florida between January 2016 and November 2017 by a certified hand therapist were reviewed retrospectively by the primary author to identify potential subjects who satisfy the inclusion and exclusion criteria and collect data pertinent to these subjects. 

Patients selection 

The inclusion criteria were males and females, age between 18-100 years old, with either surgically corrected or non-surgically treated DRFs, who participated in hand therapy between January 2016 and November 2017, with initial and discharge Quick DASH and VAS scores. The exclusion criteria were fractures related to malignancy, bilateral fractures, previous DRFs on the same side, prior limitation of ROM, cognitive impairment, worker’s compensation claims, and open fracture.

Variable of interest 

We analyzed, age, gender, initial QuickDASH scores (first-hand therapy visits) and discharge (at the last visit), initial VAS scores (first-hand therapy) and discharge (at the last visit), time from injury to hand therapy initiation (number of days between fracture diagnosis and start the hand therapy), the difference between the DASH initial and discharge and VAS initial and discharge, the total number of hand therapy visits, surgery vs non-surgery, side of the fractures.

Outcome measurements

The clinically validated QuickDASH (score range of 0-100) and VAS (score range of 0-10) scores were utilized to measure function and pain respectively. The QuickDASH is a good fit for this project as it has been shown to have test-retest reliability, as well as being simple, quick, and easy to administer [9]. The VAS scale is used to measure pain in patients with musculoskeletal dysfunction and data shows that it is a sufficiently reliable method to measure pain [10,11].

Statistical analysis

Continuous variables were summarized using mean (standard deviation (SD)) while categorical variables were reported with frequency (percentage). Change of VAS scores and QuickDASH scores between the time of initial admission and discharge of hand therapy were calculated. We performed a paired T-test to compare the means of the initial and discharge QuickDASH and VAS. All tests were two-sided with alpha level set at 0.05 for statistical significance.

## Results

Forty-nine patients were included in our study in 22 months of period time. 

Table 1 shows all the patient's characteristics. The patients’ mean age was 67.90 years, (SD 14.54), and 38 (77.6%) were female, 28 (57.1%) had a fracture on the right side, 21 (42.9%) had a fracture on the left side, 38 (77.6%) had no surgery, 11 (22.4%) had surgery.

**Table 1:** Patient characteristics

Variable	N	%
Total	49	100
Age, Mean (SD)	67.90 (14.54)	
Gender		
Male	11	22.4
Female	38	77.6
Side of the fracture		
Right	28	57.1
Left	21	42.9
Surgery		
No	38	77.6
Yes	11	22.4

Table 2 shows variables regarding hand therapy, the meantime from fracture to therapy is 32.41, (SD 24.13) days, and the mean total numbers of visits are 6.20 (SD 3.49).

**Table 2:** Hand therapy DASH: disabilities of the arm, shoulder, and hand; VAS: visual analog scale.

Variable	Mean	SD
Time to start therapy	32.41	24.13
Number of visits	6.20	3.49
DASH initial	59.27	16.93
DASH discharge	24.08	12.77
Difference in DASH	-35.18	20.09
VAS initial	3.57	1.71
VAS discharge	1.33	0.97
Difference in VAS	-2.24	2.11

Table 3, we noticed a statistically significant difference between the initial disabilities of the arm, shoulder, and hand (DASH) (59.27, SD 16.93) compared to the discharge DASH (24.08, SD 12.77) (P-value <.001); and initial VAS (3.57, SD 1.71) with a discharge VAS (1.33, SD 0.97) (P-value <.001). 

**Table 3:** DASH and VAS scores DASH: disabilities of the arm, shoulder, and hand; VAS: visual analog scale; SD: standard deviation.

	Initial	Discharge	p-value
Dash, mean (SD)	59.27 (16.93)	24.08 (12.77)	< .001
VAS, mean (SD)	3.57 (1.71)	1.33 (0.97)	< .001

## Discussion

Based on the high rate of DRFs, our study understands the importance of finding out how the affected limb can evolve with fewer sequelae, and for this, we evaluated how hand therapy is associated with positive functional outcomes. Our goal was to determine if an average of six visits to hand therapy would be sufficient to impact the difference between the initial and final DASH and VAS. We understand the importance of this study because we see all the benefits that patients could obtain since new guidelines and comparisons can be made from a new study. 

A retrospective study was conducted with 49 patients from January 2016 to November 2017 at the Mayo Clinic Florida Hand Therapy Center with a fracture of the distal radius of which met the inclusion criteria. We noticed that hand therapy positively impacts the reduction of QuickDASH and VAS scores, and the reduction was statistically significant after six visits (P< 0.001). There is conflicting data on how many hand therapy appointments are necessary. In our literature review, we found a range of 3 to 37.5 sessions from different studies [12,13]. However, the reason for which patients attend such a varied number of sessions is not clearly explained.

Hand therapy can be approached in several ways and can be performed through the home exercise program (HEP), occupational therapy (OT) or physical therapy (PT) programs, understanding that the most important is its realization [3]. 

Numerous clinical studies are demonstrating the role of hand therapy and how hand therapy impacts patients with DRFs in the short-term [5,14]. However, there is no treatment guideline that is universally agreed upon [15]. Kirby et al. in a retrospective study containing 89 patients reported that total active movement for forearm and fingers were positively associated with a greater number of hand therapy visits, while the total active motion of wrist, pain and edema which all had an insignificant correlation to increased therapy visits [4]. Valdes et al. in a retrospective study, subdivided two groups with different beginnings for hand therapy, making a comparative analysis between them, it was statistically identified that when the ROM is started within one week postoperatively (group 1), the meantime of days required of treatment with the hand therapy would be 34.79 (SD 10.9) days, with an average number of visits of 6.7 (SD 2.10) compared to group 2, which started the ROM after six weeks of postoperative, requiring a mean of 71.89 (SD 18.33) days of hand therapy, with a mean of 17.0 (SD 5.22) visits to hand therapy. Reducing the time for the initiation of hand therapy concentrates on reducing edema and consequently increasing ROM of the wrist, forearm, and fingers, as well as being able to attend to the patient's concerns to identify possible complications and intervene in an early manner [16]. 

Another important study was the systematic review comparing the management of hand therapy in uncomplicated patients and with complications secondarily, evaluating whether there are differences in the outcomes between the patients who perform a home examination program or physiotherapy performed in the clinics by professionals. It was noted that both types of hand therapy brought benefits to the patients, reducing the number of complications after DRF and better, patient return to independence with daily activities. However, the variants failed to quantify whether one approach is more efficient than the other, and the studies in this systematic review report their findings in terms of statistical significance only and do not provide the effect size. Therefore, this study can’t be generalized for all patients, since it did not accurately present the population of patients with complications and comorbidities [3]. It is difficult evaluating predictors of functional outcomes after DRFs because of differences in surgeon expertise, variability in the treatment methods, the lack of a well-defined treatment protocol, inconsistency in the follow-up times, and study protocol to collect all relevant data [17]. 

Our study is a retrospective single-center performed at the Mayo Clinic and for this reason, we consider the possibility of selection bias in the charts, we also understood that our sample for each group was small, to obtain a reliable conclusion, evaluating the differential effect of the variables evaluated. Because of our sample size, we were not able to conduct an analysis of potential factors influencing the outcomes of these patients. However, Chung et al. found no relationship between the potential predictors identified in the literature as important factors influencing the outcomes after DRF: manual dominance, gender, fracture classification [17]. A prospective data collection is important for any outcome study such as this article, to avoid issues relating to missing data and potential recall bias that can happen with retrospective studies [17]. 

To improve patient rehabilitation, we suggest that further research associate the importance of hand therapy to the treatment of DRFs and be able to subclassify the patients in a way that leads to the discovery of the most adequate continuous treatment through protocols with statistical significance of rehabilitating the patient effectively and more individualized. The high incidence of this type of injury makes important new discoveries and guidelines, understanding that there is still an information bias in the literature found.

## Conclusions

This retrospective study, conducted in a single-center, aimed to understand the importance of the early initiation of hand therapy in a single-center, with the assistance of a specialist. The reduction of the values of QuickDash and VAS evaluated clinically and statistically after six visits suggest that early rehabilitation interventions lead to improvements in pain and return to daily activity following DRF. We consider that it is difficult to develop and create a protocol when we understand that there are different methodologies for the treatment of DRF, associated with the often different characteristics of each patient, but we understand the importance of developing studies that create new guidelines for the treatment of this fracture that is commonly found in medical emergencies.
